# *Lactobacillus johnsonii* Attenuates Liver Steatosis and Bile Acid Dysregulation in Parenteral Nutrition-Fed Rats

**DOI:** 10.3390/metabo13101043

**Published:** 2023-09-29

**Authors:** Juan Xu, Yongchang Zhou, Siyang Cheng, Yuling Zhao, Junkai Yan, Ying Wang, Wei Cai, Lu Jiang

**Affiliations:** 1 Division of Pediatric Gastroenterology and Nutrition, Xinhua Hospital, School of Medicine, Shanghai Jiao Tong University, Shanghai 200092, China; xujuan66@sjtu.edu.cn (J.X.); yanjunkai@xinhuamed.com.cn (J.Y.); wangying02@xinhuamed.com.cn (Y.W.); 2 Shanghai Institute for Pediatric Research, Shanghai 200092, China; zhouyongchang@xinhuamed.com.cn; 3 Department of Pediatric Surgery, Xinhua Hospital, School of Medicine, Shanghai Jiao Tong University, Shanghai 200092, China; csy12989@rjh.com.cn (S.C.); yulingzyl@sjtu.edu.cn (Y.Z.); 4 Shanghai Key Laboratory of Pediatric Gastroenterology and Nutrition, Shanghai 200092, China

**Keywords:** gut microbiota, lipid metabolism, bile acid dysregulation, apoptosis, cholesterol 7-alpha hydroxylase

## Abstract

Parenteral nutrition (PN), a vital therapy for patients with intestinal failure, can lead to the development of parenteral nutrition-associated liver disease (PNALD). In this study, we aimed to investigate the role of *Lactobacillus johnsonii* (*L. johnsonii*) in a rat model of PNALD. Total parenteral nutrition (TPN)-fed rats were used to assess the role of *L. johnsonii* in liver steatosis, bile acid metabolism, gut microbiota, and hepatocyte apoptosis. We observed a depletion of *L. johnsonii* that was negatively correlated with the accumulation of glycochenodeoxycholic acid (GCDCA), a known apoptosis inducer, in rats subjected to TPN. *L. johnsonii* attenuated TPN-induced liver steatosis by inhibiting fatty acid synthesis and promoting fatty acid oxidation. TPN resulted in a decrease in bile acid synthesis and biliary bile secretion, which were partially restored by *L. johnsonii* treatment. The gut microbial profile revealed depletion of pathogenic bacteria in *L. johnsonii*-treated rats. *L. johnsonii* treatment reduced both hepatic GCDCA levels and hepatocyte apoptosis compared with the TPN group. In vitro, *L. johnsonii* treatment inhibited GCDCA-induced hepatocyte apoptosis via its bile salt hydrolase (BSH) activity. Our findings suggest that *L. johnsonii* protects against liver steatosis, bile acid dysregulation, and hepatocyte apoptosis in TPN-fed rats.

## 1. Introduction

Parenteral nutrition-associated liver disease (PNALD) encompasses a spectrum of liver diseases, including cholestasis, steatosis, fibrosis, and eventually cirrhosis. These conditions affect individuals with intestinal failure who depend on extended periods of parenteral nutrition (PN) [1]. The occurrence of PNALD is much greater in infants compared with adults (up to 60% vs. up to 40%), especially in premature newborns with low body weight [2]. Compared with cholestasis and portal inflammation, which can resolve in almost all patients after weaning off of PN, hepatic fibrosis and steatosis are fatal and can persist for years [3]. Therefore, PNALD represents a significant clinical challenge, and discontinuation of PN remains the only available treatment option.

Previous studies have suggested that total parenteral nutrition (TPN) leads to a significant loss of microbial diversity and alterations in the gut microbial profile in animals and infants [4,5]. Probiotic bacteria such as *Lactobacillus* and *Bifidobacterium* are commonly used in the treatment of gastrointestinal diseases such as inflammatory bowel disease, necrotizing enterocolitis, and short bowel syndrome (SBS) with controversial results [6]. *Lactobacillus johnsonii* (*L. johnsonii*) is a potentially beneficial bacterium from the genus *Lactobacillus* that may regulate metabolically related diseases [7,8]. Xin et al. [9] showed that *L. johnsonii* supplementation in mice with nonalcoholic fatty liver disease (NAFLD) resulted in improved insulin resistance, reduced fatty acid synthesis, and attenuation of mitochondrial abnormalities. Importantly, a negative correlation was reported between the duration of PN and the proportion of *Lactobacillus* in patients with SBS [10], suggesting a possible connection between PN and *Lactobacillus* depletion. In this study, we aimed to determine whether *L. johnsonii* can attenuate PNALD its the underlying mechanisms.

In this study, we revealed that the abundance of *L. johnsonii* was reduced in TPN-fed rats, a well-recognized animal model of PNALD [11]. Supplementation with *L. johnsonii* attenuated TPN-induced liver steatosis, bile acid dysregulation, and hepatocyte apoptosis. Mechanistically, *L. johnsonii* inhibited TPN-induced hepatocyte apoptosis by deconjugating glycochenodeoxycholic acid (GCDCA) [12], a known inducer of apoptosis, to chenodeoxycholic acid (CDCA) via its bile salt hydrolase (BSH) activity. The beneficial effects of *L. johnsonii* in regulating bile acid metabolism can be attributed to the increased ileal expression of *Nr1h4* (encoding for the farnesoid X receptor, FXR) and its target gene, fibroblast growth factor 19 (*Fgf19*). Our findings provide insights into *L. johnsonii* as a novel therapeutic strategy in PNALD.

## 2. Materials and Methods

### 2.1. Animals

Male Sprague–Dawley rats at the aged 4 weeks were purchased from Shanghai Laboratory Animal Center (Shanghai, China) and infused with 0.9% saline with a standard chow diet (control group) or TPN for 7 days as previously described [5,13]. Before infusion with saline or TPN, we inserted a central venous catheter (CVC) into the superior vena cava from the right jugular vein. The TPN solution underwent sterilization and was replaced daily. It included 8.5% amino acids, lipids (20% medium-chain triglyceride/long-chain triglyceride Lipofundin), 50% dextrose, electrolytes, trace elements, and vitamins, maintaining a nonprotein calorie-to-nitrogen ratio of 295:1, as previously outlined [5,13]. *L. johnsonii* was donated by Shanghai Jiaoda Onlly Co., Ltd. (China General Microbiological Culture Collection Center, No.21243). Rats in the TPN group were orally gavaged with either *L. johnsonii* containing 5 × 10^7^ CFU/day (TPN+ *L. johnsonii* group) or PBS (TPN group). All animal studies were approved by the Experimental Animal Care and Use Committee of Shanghai Jiao Tong University (No. XHEC-F-2019-003).

### 2.2. Real-Time qPCR

Bacterial genomic DNA was extracted from rat stool samples and colon mucosa as previously described [14]. TRIzol reagent was used to extract total RNA from livers according to the manufacturer’s instructions. cDNA was generated from total liver RNA using Hifair^®^ III 1st strand cDNA synthesis supermix (Yeasen, Shanghai, China), and real-time qPCR was conducted with SYBR Premix (Applied Biosystems, Waltham, MA, USA) as previously described [15]. Primer sequences are listed in Table 1. The relative mRNA expression for rat genes and *L. johnsonii* was normalized to *18S* and *16S*, respectively.

### 2.3. Western Blot Analysis

Protein levels of cleaved caspase-3 in rat livers were determined by Western blot analysis. Proteins extracted from livers were subjected to SDS-PAGE gels and electrotransferred to polyvinylidene fluoride (PVDF) membranes. Nonspecific binding sites were blocked in 5% bovine serum albumin at room temperature for 1 h. Membranes were subsequently probed with primary antibodies at 4 °C overnight, then incubated with the appropriate peroxidase-conjugated secondary antibody. Cleaved caspase-3 monoclonal antibody (1:1000; Cell Signaling Technology, Boston, MA, USA) and Cyp7a1 antibody (1:1000; Shenggong BBI Life, Shanghai, China) were used as primary antibodies, and horseradish-peroxidase-conjugated anti-rabbit IgG (1:5000; Abclonal, Wuhan, China) was used as a secondary antibody. Densitometry of immunoblot analysis was performed using ImageJ software as previously described [14].

### 2.4. Bile Acid Measurement

Liver and serum bile acids were measured by a liquid chromatography tandem mass spectrometry (LC-MS) system (BioNovoGene, Suzhou, China). Briefly, blood samples were obtained and centrifuged to collect serum for analysis. For liver tissues, approximately 50 mg of lyophilized, homogenized tissue was weighed and grounded with methanol to precipitate protein. After vortexing and centrifuging at 4 °C, the supernatant was mixed with water and filtered before being analyzed [16]. A total of 38 bile acids were quantified in both liver and serum.

### 2.5. Bacterial DNA Extraction and 16S rDNA Sequencing

DNA from rat feces was extracted using a TIANamp stool DNA kit (Tiangen, Beijing, China) following the manufacturer’s instructions. The V3-V4 regions of the 16S ribosomal DNA gene were amplified and purified for sequencing using an Illumina MiSeq instrument (Illumina, San Diego, CA, USA). Raw sequencing data was decomposed and filtered for quality control. Operational taxonomic units (OTUs) were clustered at 97% similarity, and taxonomic classification was performed using UCLUST software [17] with the SILVA database (V128, http://www.arb-silva.de, accessed on 16 December 2019) [18]. The functional potential of microbial communities was predicted based on 16S rRNA gene sequencing data by PICRUST2 (Phylogenetic Investigation of Communities by Reconstruction of Unobserved States) [19]. Raw sequence reads for *L. johnsonii* were deposited in the NCBI Sequence Read Archive (SRA) under Bioproject PRJNA1004452.

### 2.6. L. johnsonii Whole-Genome Sequencing

Bacterial genomic DNA was extracted from *L. johnsonii*, detected by agarose gel electrophoresis, and quantified by a Qubit 2.0 fluorometer. Libraries for nanopore sequencing were constructed with an insert size of 10 kb. DNA was fragmented to a size of 350 bp to construct libraries for the Illumina NovaSeq platform. The whole genome of *L. johnsonii* was sequenced on the Nanopore PromethION platform and Illumina NovaSeq PE150 at Beijing Novogene Bioinformatics Technology Co., Ltd (Beijing, China). PE150 data and nanopore data were assembled and subjected to genome component prediction. For bacteria, the GeneMarkS program was used to retrieve the related coding gene. Raw sequence reads for *L. johnsonii* were deposited in the NCBI (SRA) associated with Bioproject PRJNA990471.

### 2.7. Liver Histology and Biochemical Assay

Formalin-fixed liver tissue was embedded in paraffin and cut to a thickness of 5 μm for H&E staining. To assess lipid accumulation, liver sections were embedded into OCT compound, sliced to a thickness of 8 μm, and stained with Oil red O (Sigma-Aldrich, St.Louis, USA) as previously described [13,15]. Cell death was assessed by terminal deoxynucleotide transferase-mediated dUTP nick-end labeling (TUNEL) staining using a fluorescein (FITC) TUNEL cell apoptosis detection kit (Servicebio, Wuhan, China).

### 2.8. Biochemical Assay

Aspartate aminotransferase (AST), alanine aminotransferase (ALT), total bile acids, and direct bilirubin in serum were measured using commercially available detection kits (Nanjing Jiancheng Bioengineering Institute, Nanjing, China). Hepatic triglyceride levels were measured using a triglyceride assay kit (GPO-POD; Applygen Technologies Inc., Beijing, China) as previously described [14]. Hepatic cholesterol levels were measured using a tissue total cholesterol kit (Applygen Technologies Inc., Beijing, China). The final concentrations of triglyceride and cholesterol were corrected according to the protein concentration.

### 2.9. BSH Activity Analysis and Apoptosis Assay

*L. johnsonii* proteins were prepared using the sonication method. Incubation was carried out in 0.1 M sodium phosphate containing 0.1 mg/mL *L. johnsonii* protein, 10 mM GCDCA, and 100 μM caffeic acid phenethyl ester (CAPE, BSH inhibitor) as previously described [20]. The mixtures were incubated at 37 °C for 90 min, and the reactions were subsequently stopped by 15% (*w/v*) trichloroacetic acid (1:1 ratio). The supernatants were collected after centrifuging for GCDCA quantification by LC-MS and in vitro experiments.

HepG2 cells were cultured with Dulbecco’s Modified Eagle Medium, which was supplemented with 10% fetal bovine serum and 1% penicillin/streptomycin. Then, 1% bacterial supernatant was added to cell culture medium and incubated for 6 h. The final concentration for GCDCA in the cell culture medium was 100 μM. Caspase 3/7 activity was measured using an Apo-ONE homogeneous caspase-3/7 assay kit (Promega, Madison, WI, USA).

### 2.10. Statistical Analysis

All data are expressed as mean ± SEM unless otherwise stated. Comparisons between two groups were performed using Student’s *t* test or Mann–Whitney–Wilcoxon rank-sum test for highly skewed distributions. The significance of multiple groups was evaluated using one-way ANOVA with Tukey’s post hoc test. Spearman’s correlation was performed for correlation analysis. Statistical analysis was performed with GraphPad Prism (V.8.4.2). A value of *p* < 0.05 was considered statistically significant.

## 3. Results

### 3.1. Reduction in L. johnsonii Is Associated with an Increased GCDCA Level in TPN-Fed Rats

**Figure 1:**
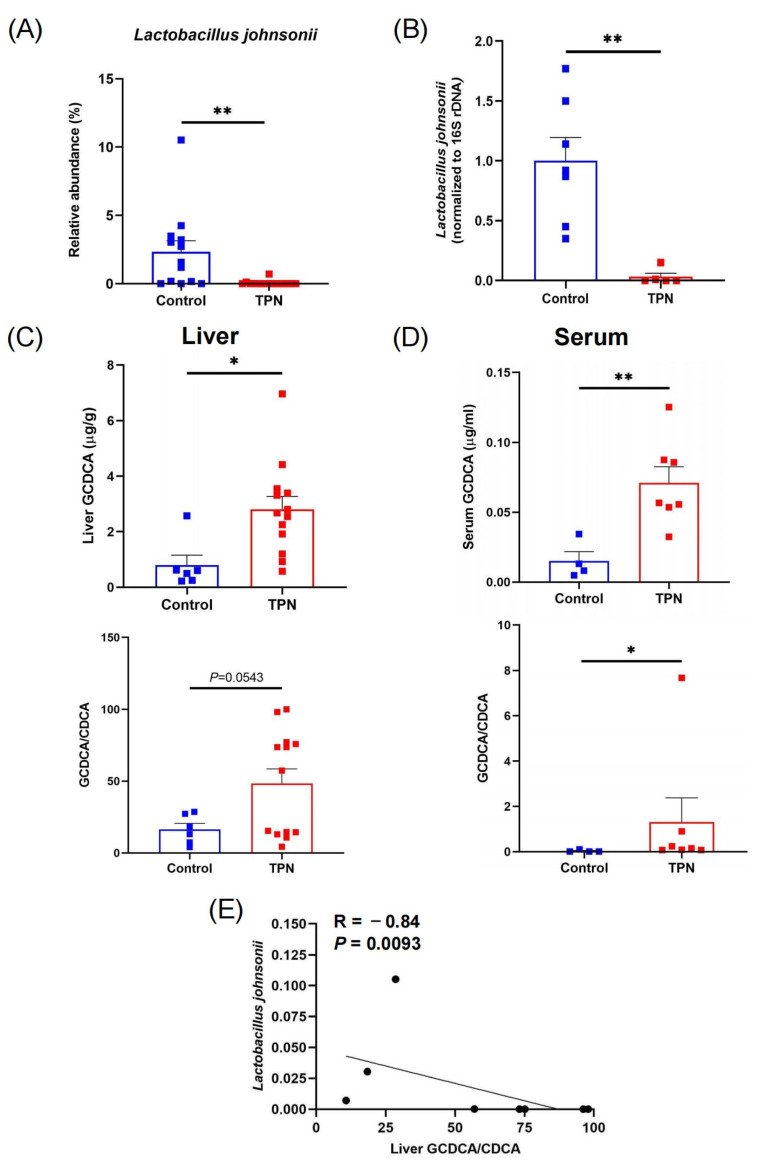
TPN induces a reduction in *L. johnsonii* and an increase in GCDCA levels in rats. (**A**) Relative abundance of *L. johnsonii* in rat fecal samples from the control group and TPN group assessed by 16S rDNA sequencing. (**B**) *L. johnsonii* levels in rat fecal samples from the control group and TPN group assessed by qPCR. (**C**,**D**) The concentrations of GCDCA and the ratios of GCDCA/CDCA in rat liver (**C**) and serum (**D**). (**E**) Spearman’s correlation between liver GCDCA/CDCA ratio and the relative abundance of *L. johnsonii*. * *p* < 0.05, ** *p* < 0.01. n = 6–13 per group.

Using 16S rDNA sequencing [5], we observed a significant decrease in the abundance of the probiotic species *L. johnsonii* in TPN-fed rats (Figure 1A). To validate these findings, we performed qPCR analysis on fecal DNA samples from rats, which further confirmed a substantial reduction in *L. johnsonii* in the TPN group compared with controls (Figure 1B). Since it is well known that *Lactobacillus* species possess BSH enzymes that mediate bile acid deconjugation [21], we next sought to investigate whether the reduction in *L. johnsonii* could potentially lead to a decrease in specific bile acid deconjugation. By evaluating bile acid profiles, we observed significant increases in GCDCA levels and GCDCA/CDCA ratios in both livers (Figure 1C) and serum (Figure 1D) from TPN-fed rats compared with controls. Correlation analysis showed that the liver GCDCA/CDCA ratio was negatively correlated with the relative abundance of *L. johnsonii* (Figure 1E), suggesting a possible link between the TPN-induced reduction in *L. johnsonii* and GCDCA accumulation. Since GCDCA is a strong inducer of apoptosis [22], these data indicate that the TPN-induced reduction in *L. johnsonii* may contribute to GCDCA accumulation, further leading to hepatocyte apoptosis and liver injury.

### 3.2. L. johnsonii Treatment Alleviates Hepatic Steatosis in TPN-Fed Rats

**Figure 2:**
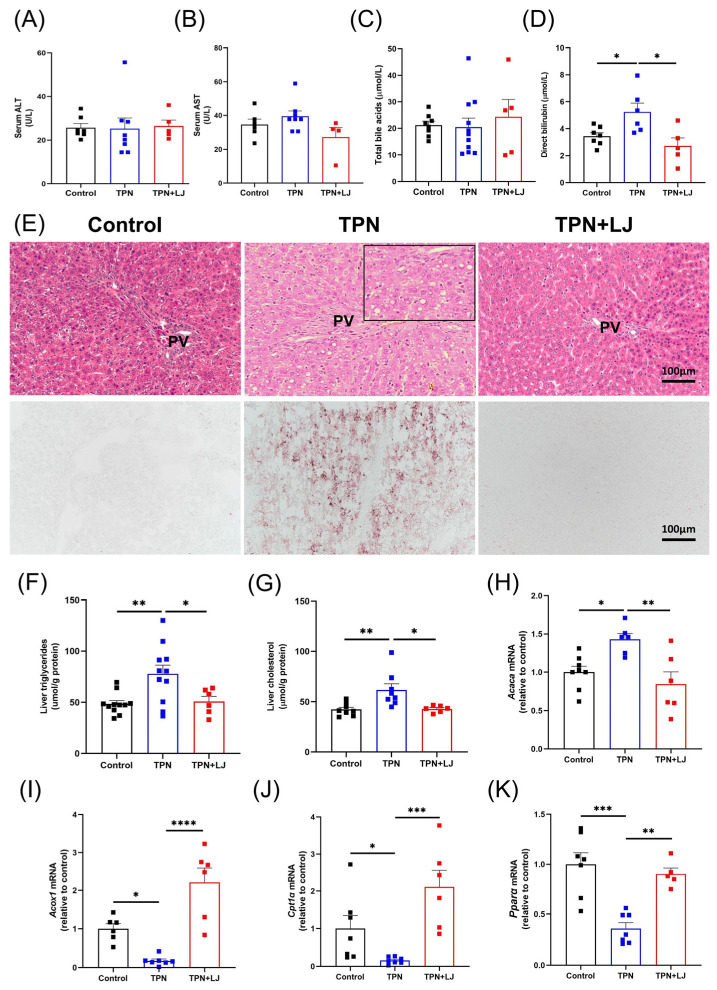
*L. johnsonii* treatment attenuates TPN-induced hepatic steatosis in rats. (**A**–**D**) Serum levels of ALT (**A**), AST (**B**), total bile acids (**C**), and direct bilirubin (**D**) in rats from the control, TPN, and TPN + *L. johnsonii* (LJ) groups. (**E**) Representative images of H&E (200 × magnification) and oil red O (200× magnification) staining on liver sections. (**F**,**G**) Liver triglycerides (**F**) and liver cholesterol (**G**) levels in rats from different groups. (**H**–**K**) The mRNA levels of *Acaca* (**H**)*, Acox1* (**I**)*, Cpt1α* (**J**), and *Pparα* (**K**) in rats from different groups. * *p* < 0.05, ** *p* < 0.01, *** *p* < 0.001, **** *p* < 0.0001. n = 5–11 per group. PV, portal vein.

In our previous studies, we established that TPN infusion for 7 days led to the manifestation of liver steatosis and perturbation in bile acid metabolism in rats [5,13,23]. We first evaluated body weight (Supplementary Figure S1A) and the percentage of liver/body weight (Supplementary Figure S1B). In rats from different groups, we observed a significant decrease in body weight in the TPN group compared with the control group, while TPN and TPN + LJ exhibited no difference (Supplementary Figure S1A). The percentage of liver/body weight ratio exhibited no difference among all groups (Supplementary Figure S1B). We next assessed the impact of *L. johnsonii* in mitigating TPN-induced liver injury. Consistent with previous findings [5], serum levels of ALT (Figure 2A), AST (Figure 2B), and total bile acids (Figure 2C) were not affected by TPN or TPN + *L. johnsonii* treatment compared with controls. Direct bilirubin was significantly increased by TPN, while was reduced by *L. johnsonii* treatment (Figure 2D). Notably, treatment with *L. johnsonii* effectively attenuated TPN-induced hepatic steatosis, as evidenced by the histological analysis of liver sections stained with H&E and oil red O (Figure 2E), liver triglycerides (Figure 2F), and liver cholesterol levels (Figure 2G). Cytosolic acetyl-CoA carboxylase 1 (*Acaca*), a gene associated with fatty acid synthesis, showed significant upregulation in response to TPN, which was effectively downregulated upon *L. johnsonii* treatment (Figure 2H). The expression levels of genes associated with fatty acid oxidation (*Acox1*, *Cpt1α*, and *Pparα*) were downregulated by TPN, whereas their expression was upregulated after *L. johnsonii* treatment (Figure 2I–K). These data suggest that *L. johnsonii* treatment attenuated liver steatosis in TPN-fed rats by decreasing fatty acid synthesis and increasing fatty acid oxidation.

### 3.3. L. johnsonii Treatment Attenuates TPN-Induced Dysregulation of Bile Acid Metabolism

**Figure 3:**
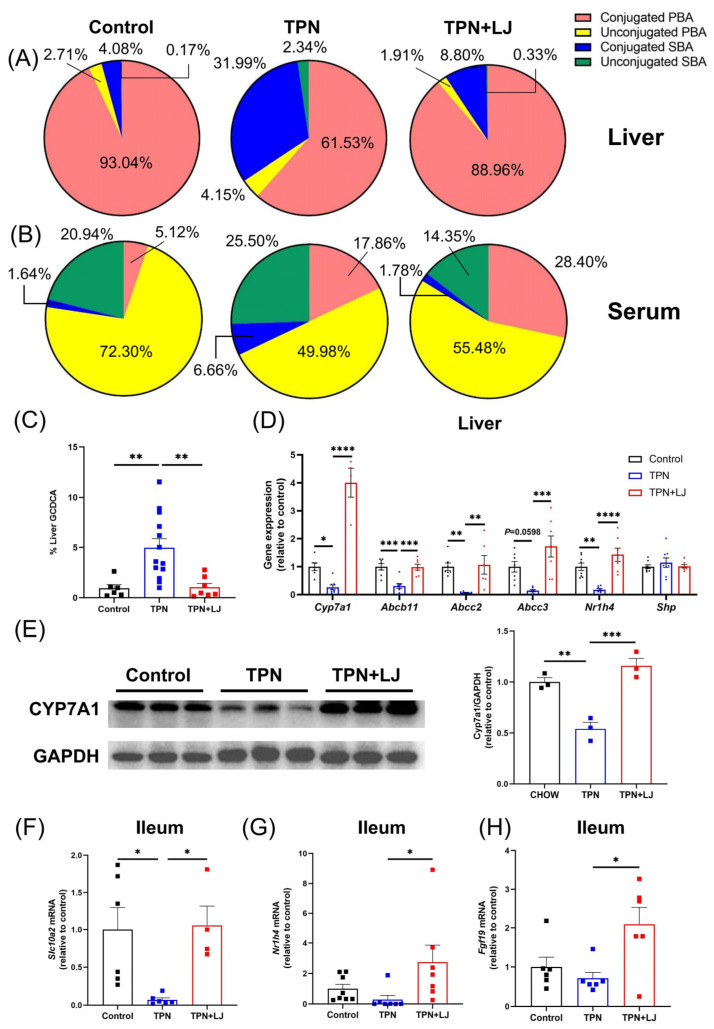
Dysregulation of bile acid metabolism is improved by *L. johnsonii* treatment in TPN-fed rats. (**A**,**B**) The bile acid profiles in the liver (**A**) and serum (**B**) of rats from the control, TPN, and TPN + *L. johnsonii* (LJ) groups. (**C**) Percentage of GCDCA levels in the liver from different groups. (**D**) The hepatic expression of *Cyp7a1, Abcb11, Abcc2, Abcc3, Nr1h4,* and *Shp* in rats from different groups. (**E**) Western blot analyses and densitometric quantification of CYP7A1 in liver. (**F**,**H**) The ileal expression of *Slc10a2* (**F**), *Nr1h4* (**G**), and *Fgf19* (**H**) in rats from different groups. * *p* < 0.05, ** *p* < 0.01, *** *p* < 0.001, **** *p* < 0.0001. n = 3–13 per group.

Next, we analyzed the effects of *L. johnsonii* treatment on the bile acid profile (refer to Table 2 for statistics and Supplementary Table S1 for details of bile acid composition) and the expression of genes associated with bile acid metabolism. In the liver, the TPN-induced reduction in conjugated primary bile acids (PBA) was reversed by *L. johnsonii* treatment. Both conjugated and unconjugated secondary bile acids (SBA) were expanded in the TPN group, while the proportions were reduced by *L. johnsonii* treatment (Figure 3A). In serum, the proportion of unconjugated PBA was significantly reduced by TPN, while the proportion was moderately increased by *L. johnsonii* treatment. The percentage of conjugated PBA was significantly increased by TPN compared with controls, while *L. johnsonii* treatment exhibited no change. Interestingly, the proportion of conjugated SBA was increased in the TPN group but reduced following *L. johnsonii* treatment (Figure 3B). Importantly, the percentage of GCDCA in the liver was significantly increased in the TPN group, and treatment with *L. johnsonii* resulted in a notable reduction (Figure 3C). These results indicate that TPN induced a notable alteration of the liver bile acid profile and hepatic accumulation of GCDCA, both of which were reversed by *L. johnsonii* treatment.

Next, we analyzed genes associated with bile acid metabolism. TPN administration resulted in a significant reduction in hepatic expression of cholesterol 7α-hydroxylase (*Cyp7a1*) (Figure 3D,E) accompanied by an increase in hepatic cholesterol levels (Figure 2G), both of which were reversed by *L. johnsonii* treatment. These data suggest that TPN-induced inhibition of bile acid synthesis was attenuated by *L. johnsonii*. In addition, hepatic expression of genes associated with bile secretion and exportation, including *Abcb11* (Bile salt export pump, Bsep), *Abcc2* (Multidrug resistance-associated protein 2, Mrp2), and *Abcc3* (multidrug resistance-associated protein 3, Mrp3), was significantly inhibited by TPN, while their expression was upregulated by *L. johnsonii* treatment (Figure 3D). The ileal expression of *Slc10a2*, a transporter responsible for the uptake of conjugated bile acids at the terminal ileum, was significantly downregulated by TPN but promoted by *L. johnsonii* treatment, indicating a possible role of *L. johnsonii* in promoting bile acid reuptake from the ileum (Figure 3F). Collectively, our findings suggest that TPN significantly inhibits bile acid synthesis, biliary bile salt secretion, and bile acid reuptake, all of which were improved by *L. johnsonii* treatment.

Hepatic *Cyp7a1* expression is known to be suppressed by farnesoid X receptor (Fxr, encoded by *Nr1h4*)-induced expression of fibroblast growth factor (Fgf) 19 in the terminal ileum [24]. Interestingly, the expression of ileal *Nr1h4* and *Fgf19* in the ileum was unaffected by TPN, while *L. johnsonii* significantly upregulated their expression levels (Figure 3G,H), indicating that the negative feedback regulation of bile acid synthesis is nonfunctional in TPN-fed rats. Since Cyp7a1 is additionally regulated by hepatic Fxr, we next evaluated the hepatic expression of *Nr1h4* and its target gene, *Shp*. TPN significantly inhibited the hepatic expression of *Nr1h4* compared with controls, and its expression was significantly promoted by *L. johnsonii* treatment (Figure 3D). However, the hepatic expression of *Shp* was not affected (Figure 3D). Taken together, these data suggest that despite reduced bile acid synthesis, TPN-fed rats appeared to be resistant to Fxr and Fgf19 regulation.

### 3.4. L. johnsonii Modulates the Gut Microbiota of TPN-Fed Rats

**Figure 4:**
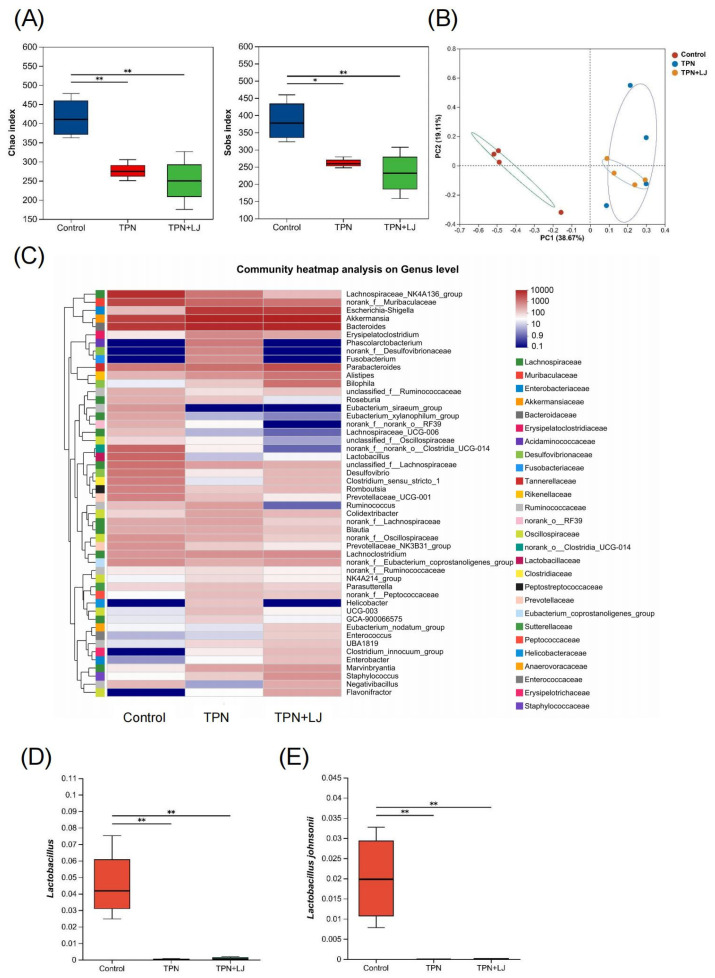
*L. johnsonii* modulates the gut microbial profile of TPN-fed rats. (**A**) Alpha diversity (Chao index and Sobs index) of fecal microbiota OTU at various taxonomic ranks in the control, TPN, and TPN + *L. johnsonii* (LJ) groups. (**B**) Principal coordinate analysis (PCoA) of β-diversity based on the Bray–Curtis dissimilarity matrix of OTU-level compositional profiles. (**C**) Community heatmap analysis of the gut microbiota at the genus level. (**D**,**E**) The relative abundance of *Lactobacillus* (**D**) and *L. johnsonii* (**E**) in fecal samples from different groups. * *p* < 0.05, ** *p* < 0.01. n = 4 per group.

To investigate the effects of *L. johnsonii* on the gut microbiota, we performed 16S rDNA gene sequencing on rat stool samples. The microbial diversity was significantly reduced by TPN, as shown by the Chao and Sobs indices, while there was no difference between the TPN + *L. johnsonii* and TPN groups (Figure 4A). Similarly, the β-diversity of the control group was distinct from that of the TPN group, while the TPN + *L. johnsonii* and TPN groups showed a similar pattern (Figure 4B). Next, we performed heatmap analysis to reveal the differences at the genus level. Interestingly, some genera with species of pathogenic potential, such as *Fusobacterium* and *Helicobacter*, exhibited decreased abundances in rats treated with *L. johnsonii* compared with the TPN group (Figure 4C). Both *Lactobacillus* and *L. johnsonii* were significantly reduced by TPN, and *L. johnsonii* supplementation did not affect their abundance (Figure 4D,E). Taken together, these results suggest that although *L. johnsonii* did not change the overall gut microbial composition, it could potentially deplete gut pathogens.

### 3.5. L. johnsonii Treatment Reduces Hepatocyte Apoptosis by Deconjugating GCDCA

**Figure 5:**
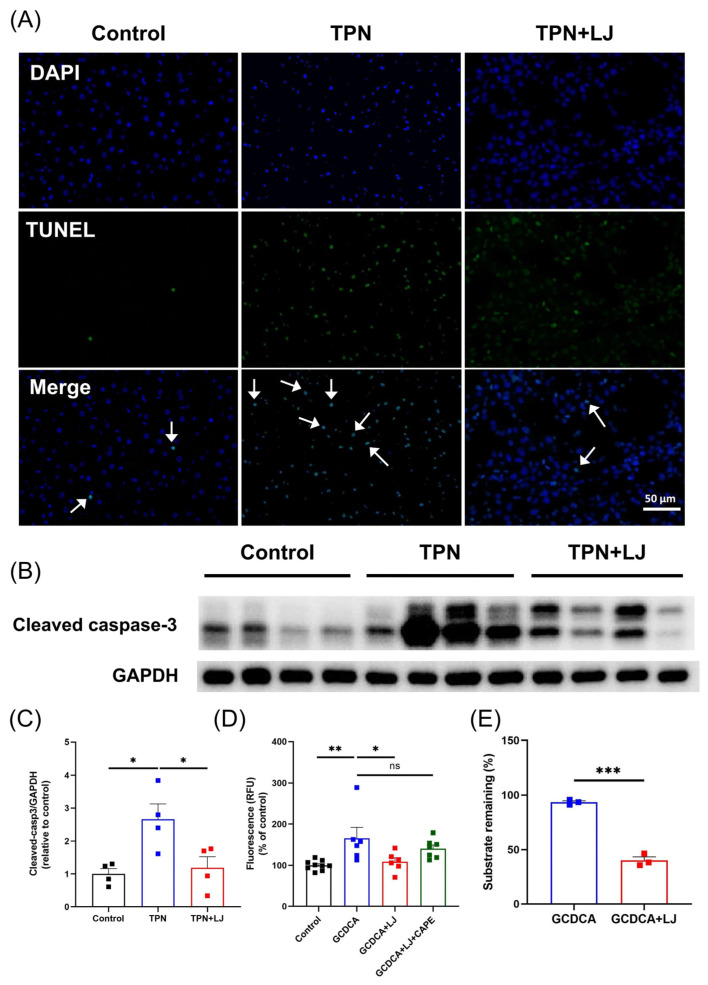
*L. johnsonii* treatment attenuates TPN-induced hepatocyte apoptosis by deconjugating GCDCA. (**A**) TUNEL staining on liver sections of rats in the control, TPN, and TPN + *L. johnsonii* (LJ) groups at 400× magnification. White arrows indicate TUNEL positive cells. (**B**,**C**) Western blot analyses and densitometric quantification of cleaved caspase-3 in the liver. (**D**) The caspase 3/7 activity of HepG2 cells after treatment with the supernatants from the control, GCDCA, GCDCA + LJ protein, and GCDCA + LJ + caffeic acid phenethyl ester (CAPE) groups. (**E**) The percentage of remaining GCDCA in culture medium after GCDCA or GCDCA + LJ group treatment. * *p* < 0.05, ** *p* < 0.01, *** *p* < 0.001; ns: not significant. n = 3–9 per group.

We previously showed that TPN-induced hepatocyte apoptosis in rats [25] and humans with PNALD [26] may contribute to the development of PNALD. GCDCA is thought to induce hepatocyte apoptosis by Fas death receptor-dependent signaling independent of Fas ligand [12,27]. Considering that hepatic GCDCA accumulation was attenuated by *L. johnsonii*, we next evaluated whether hepatocyte apoptosis was reduced. As we expected, hepatocyte apoptosis was aggravated by TPN and was alleviated by *L. johnsonii* treatment, as shown by TUNEL staining (Figure 5A) and Western blotting on cleaved caspase-3 (Figure 5B,C). To evaluate whether *L. johnsonii* with BSH activity was responsible for the reduction of GCDCA by directly deconjugating to CDCA in vitro, we performed whole-genome sequencing on *L. johnsonii* to characterize its genome component and function. As we expected, *L. johnsonii* was predicted to encode for the *bsh* gene (KEGG orthologue K01442), suggesting a possible BSH activity that may contribute to GCDCA deconjugation (Table 3). Importantly, *L. johnsonii* significantly inhibited GCDCA-induced apoptosis in HepG2 cells, while the effect was abolished by coculturing with BSH inhibitor CAPE (Figure 5D). Lastly, we treated HepG2 cells with 100 μM of GCDCA, along with *L. johnsonii* (4 × 10^4^ CFU/well). After 6 h, the remaining GCDCA concentration was measured by LC-MS/MS. As we expected, we observed a significant reduction in GCDCA levels in the group treated with GCDCA + *L. johnsonii*, suggesting that *L. johnsonii* could directly deconjugate GCDCA to CDCA (Figure 5E). Taken together, our results suggest that *L. johnsonii* inhibits GCDCA-induced hepatocyte apoptosis through its BSH activity.

## 4. Discussion

In this study, we demonstrated, for the first time, that oral administration of *L. johnsonii* attenuated liver steatosis, bile acid dysregulation, and hepatocyte apoptosis in TPN-fed rats. Through in vitro experiments, we found that *L. johnsonii* inhibited GCDCA-induced hepatocyte apoptosis by deconjugating it to CDCA.

Consistent with previous findings from Koelfat et al. [11] and Wang et al. [5], we showed that TPN administration for 7 days induced moderate liver steatosis, dysregulation of bile acids, and gut dysbiosis in rats. However, levels of ALT, AST, and total bile acids remained unaffected. After *L. johnsonii* treatment, liver steatosis was attenuated as a result of decreased fatty acid synthesis and increased fatty acid oxidation. We previously showed that the expression of hepatic *Cpt1α*, an enzyme involved in fatty acid oxidation, was significantly suppressed in both piglet and rat models of PNALD [23]. Here, we found that *L. johnsonii* promoted the expression of *Cpt1α*, while the underlying mechanisms remain to be elucidated. A previous study conducted by Jang et al. [28] reported a novel mechanism through which *Lactobacillus rhamnosus GG* (LGG) improved liver steatosis induced by HFD in mice. By using radioactive tracers and LC-MS methods, they showed that LGG and hosts competed for fatty acid absorption in the intestine, resulting in hepatic lipid accumulation and decreased body weight. Due to their ability to ferment sugars, *Lactobacillus* bacteria are known to produce lactate. Another study conducted by Ritze et al. [29] showed that LGG facilitated the growth of butyrate-producing bacteria and enhanced intestinal barrier function. Compared with LGG, *L. johnsonii* is less studied in metabolic liver diseases. Future studies could aim to unravel the specific roles of *L. johnsonii*-derived metabolites in PNALD, exploring their mechanisms in both preclinical and clinical settings.

Previous studies have reported that PN disrupts the enterohepatic circulation, resulting in bile “stasis” and subsequent cholestatic liver injury [30,31]. The onset of PN-induced cholestasis was associated with alterations in bile salt synthesis and transport [11]. Here, we showed that TPN administration resulted in alterations to the bile acid composition and metabolism. The most notable change observed in the TPN group was the reduced proportion of conjugated PBA in the liver, which was restored by *L. johnsonii*. In line with previous findings, Zhan et al. [32] analyzed the liver bile acid profile in a mouse model of PNALD and observed a significant reduction in PBA, particularly taurine-conjugated bile acids, such as taurochenodeoxycholic acid (TCDCA), tauroα-muriholic acid (T-α-MCA), tauroβ-muriholic acid (T-β-MCA), and tauroursodeoxycholic acid (TUDCA), following TPN administration. In contrast to PBA, the proportion of SBA, particularly conjugated SBA, was significantly increased in the TPN group. Our study indicated a reduction in de novo bile acid synthesis in response to TPN, aligning with previous findings of Koelfat et al. [11], who reported decreased bile salt synthesis in TPN-fed rats for up to 14 days. Furthermore, both our studies revealed a decrease in biliary bile secretion induced by TPN, which was ameliorated by *L. johnsonii* treatment. These findings collectively suggest a potential beneficial role of *L. johnsonii* in mitigating TPN-induced bile stasis.

It is widely recognized that Cyp7a1 is negatively regulated by ileal Fxr/Fgf19 and hepatic Fxr/Shp signaling. In the present study, we observed that ileal Fxr/Fgf19 signaling remained unaffected by TPN, while hepatic Fxr was suppressed, accompanied by a reduction in hepatic Cyp7a1 expression. Interestingly, *L. johnsonii* treatment promoted both ileal Fxr/Fgf19 signaling and Cyp7a1 expression, suggesting that the impaired regulation of Fxr caused by TPN was not restored by *L. johnsonii*. Furthermore, the *L. johnsonii* treatment group exhibited an elevated proportion of taurocholic acid (TCA) and CDCA in the serum (Table S1). Both TCA and CDCA are known Fxr agonists, and their increased levels may contribute to the upregulation of Fxr/Fgf19 expression observed in this study. The inability of Fxr activation to effectively inhibit bile acid synthesis has been documented in both animal models and humans with NAFLD/NASH [33,34,35,36]. Unfortunately, the underlying mechanisms that contribute to the nonresponsiveness of Cyp7a1 to Fxr activation remain crucial yet poorly understood.

An increasing body of evidence suggests that PN leads to increased intestinal permeability and gut dysbiosis, which could lead to the development of PNALD [37]. There is bidirectional communication between the gut and liver through the portal vein, biliary tract, and systemic circulation. A previous study conducted by Dehemri et al. [38] showed that TPN induced a decrease in intestinal epithelial cell proliferation and an increase in apoptosis in mice. Eventually, gut microbiota and microbial products (such as lipopolysaccharides) can enter the liver via the portal vein to induce liver injury. In our study, we proposed that *L. johnsonii* could deconjugate GCDCA to CDCA, thereby reducing hepatocyte apoptosis and liver injury. It also exhibited beneficial effects by promoting ileal FXR/FGF19 signaling. Future studies should be performed to investigate the role of *L. johnsonii* in intestinal permeability. Using 16S rDNA sequencing, we observed a significant reduction in microbial diversity in the TPN group, while there was no difference between the TPN and TPN + *L. johnsonii* groups. Meanwhile, we found that TPN increased the abundance of two potential pathogenic genera, *Fusobacterium* and *Helicobacter*, which may contribute to the pathogenesis of PNALD. A previous study conducted by Cao et al. [39] showed that *Fusobacterium nucleatum* promoted liver damage in acute liver failure by inhibiting NAD^+^ and the NAD^+^-dependent SIRT1/AMPK signaling pathway. In addition, *Helicobacter pylori* infection has been linked to the development of both acute and chronic liver diseases, such as viral hepatitis [40], NAFLD [41], and HCC [42]. Consistent with our findings, PN has been shown to promote the growth of opportunistic pathogens, including *Vibrio*, *Escherichia. coli*, *Salmonella*, *Yersinia*, and *Helicobacter* [6,43]. These findings suggest that *L. johnsonii* may attenuate PNALD progression by modulating the gut microbiota. Further investigations could explore the potential impact of prolonged *L. johnsonii* supplementation on the gut microbiota in the context of PNALD.

As the most extensively studied form of regulated cell death in PNALD, increased hepatocyte apoptosis has been consistently reported in preclinical studies, as well as in patients with PNALD [44,45,46]. However, there is currently a lack of studies investigating the role of apoptosis inhibitors in PNALD. Consistently, we demonstrated that the depletion of *L. johnsonii* led to a reduction in GCDCA deconjugation, which subsequently triggered hepatocyte apoptosis and contributed to the development of PNALD. *Lactobacillus* is a genus known for its bile salt hydrolase (BSH) activity and bile resistance, which contribute to its ability to persist and colonize the gut [47]. Using whole genome sequencing, we identified three BSH genes in the genome of *L. johnsonii*. Furthermore, our in vitro experiments demonstrated that *L. johnsonii* possesses the ability to deconjugate GCDCA. Our study provides valuable insights into the mechanisms underlying PN-induced gut dysbiosis and the promotion of hepatocyte apoptosis. These findings open up new possibilities for the development of therapeutic approaches to manage PNALD.

This work is subject to several important limitations. First, although most patients who received TPN have SBS (either congenital or acquired), the rats retained the entirety of their bowel. Therefore, careful investigation is critical in translation to human PNALD patients. Second, although hepatic GCDCA was reduced after *L. johnsonii* treatment, serum GCDCA remained unaffected. Further investigations to evaluate the effects of *L. johnsonii* on the bile acid pool are warranted.

In summary, to the best of our knowledge, this is the first study to demonstrate the protective effects of *L. johnsonii* in a rat model of PNALD. *L. johnsonii* supplementation can protect against TPN-induced liver steatosis, bile acid dysregulation, and hepatocyte apoptosis. Mechanistically, *L. johnsonii* attenuates TPN-induced hepatocyte apoptosis by directly deconjugating GCDCA via its BSH activity. These findings may facilitate the development of therapeutic strategies utilizing probiotics to prevent PNALD pathogenesis.

## Figures and Tables

**Table 1:** Primer sequences for quantitative PCR.

Gene Name	Forward Sequence	Reverse Sequence
*Abcb11*	CTGCCAAGGATGCTAATGCA	CGATGGCTACCCTTTGCTTCT
*Abcc2*	TCGAGAGAGGCTGACCATCA	TCTGCCCTATGCTCAGGTTG
*Abcc3*	GCCTTACAGGTGACCTTGAGTT	CGGTACCGCACCGAATAGTT
*Acaca*	CCCAGAGATGTTTCGGCAGTCAC	GTCAGGATGTCGGAAGGCAAAGG
*Acox1*	AGTCTGAAATCAAGCAAAGC	CATTAATTCGAAGGTAGGTCTC
*Cpt1α*	TAGGACAGGCAGAAAATTGC	CAGTAGGAGCCGATTCAAAA
*Cyp7a1*	TGCCTTCTGTTACCGAGTGATGT	ACCGGCAGGTCATTCAGTTGCACT
*Fgf19*	ATACGGGCTGATTCGCTACT	GCTGGTCCGTGGATTCAAAG
*Nr0b2*	CGATCCTCTTCAACCCAGATG	AGGGCTCCAAGACTTCACACA
*Nr1h4*	GAAACTGAACATCGGGGTTAT	CGGCGGAGATTTTCAATAAG
*Ntcp*	CAAACCTCAGAAGGACCAAACA	GTAGGAGGATTATTCCCGTTGTG
*Pparα*	AATGCAATCCGTTTTGGAAG	TTGGCCAGAGATTTGAGGTC
*Slc10a2*	TGGTGTAGACGAAGAGGCAA	GCCTATTGGATAGATGGCGA
*18S*	ACGGAAGGGCACCACCAGGA	CACCACCACCCACGGAATCG
*16S*	GTGSTGCAYGGYTGTCGTCA	ACGTCRTCCMCACCTTCCTC
*L. johnsonii*	AGAGAGAAACTCAACTTGAAATA	CCTTCATTAACCTTAACAGTTAA

Notes: *L. johnsonii*, *Lactobacillus johnsonii*.

**Table 2:** Statistics (adjusted *p* value) for bile acid composition.

	Control vs. TPN	TPN vs. TPN + LJ
Liver		
% unconjugated PBA/TBA	0.4071	0.1041
% conjugated PBA/TBA	<**0.0001**	<**0.0001**
% unconjugated SBA/TBA	**0.0369**	**0.0429**
% conjugated SBA/TBA	<**0.0001**	<**0.0001**
Serum		
% unconjugated PBA/TBA	**0.0374**	0.7035
% conjugated PBA/TBA	0.6261	0.6387
% unconjugated SBA/TBA	0.4235	0.1033
% conjugated SBA/TBA	**0.0227**	**0.0478**

Notes: Bold font indicates significance (*p* value < 0.05). TPN, total parenteral nutrition; LJ, *Lactobacillus johnsonii*; PBA, primary bile acid; SBA, secondary bile acid; TBA, total bile acid.

**Table 3:** Genes in *Lactobacillus johnsonii* with bile salt hydrolase activity.

Gene ID	Identity	E Value	KO ID	KO Definition
LJ4_GM000177	99.1	9.40 × 10^−185^	K01442	choloylglycine hydrolase
LJ4_GM000897	100	3.10 × 10^−191^	K01442	choloylglycine hydrolase
LJ4_GM001072	99.4	1.20 × 10^−187^	K01442	choloylglycine hydrolase

## Data Availability

The datasets analyzed during the current study are available from the corresponding author upon reasonable request. The data are not publicly available due to privacy.

## Supplementary Materials

The following supporting information can be downloaded at: https://www.mdpi.com/article/10.3390/metabo13101043/s1, Figure S1: The body weight and ratio of liver weight to body weight of rats from the control, TPN, and TPN + *L. johnsonii* (LJ) groups. Table S1: Summary of liver and serum bile acid composition in rats from the control, TPN, and TPN + *L. johnsonii* (LJ) groups.
