# Supplementary material for: Lactobacillus johnsonii Attenuates Liver Steatosis and Bile Acid Dysregulation in Parenteral Nutrition-Fed Rats
Source: Metabolites. 2023 Sep 29;13(10):1043. doi: 10.3390/metabo13101043 (PMC10608838; doi:10.3390/metabo13101043)
Supplement: Supplementary file 1 [file metabolites-13-01043-s001.zip › metabolites-2587441-supplementary.pdf]

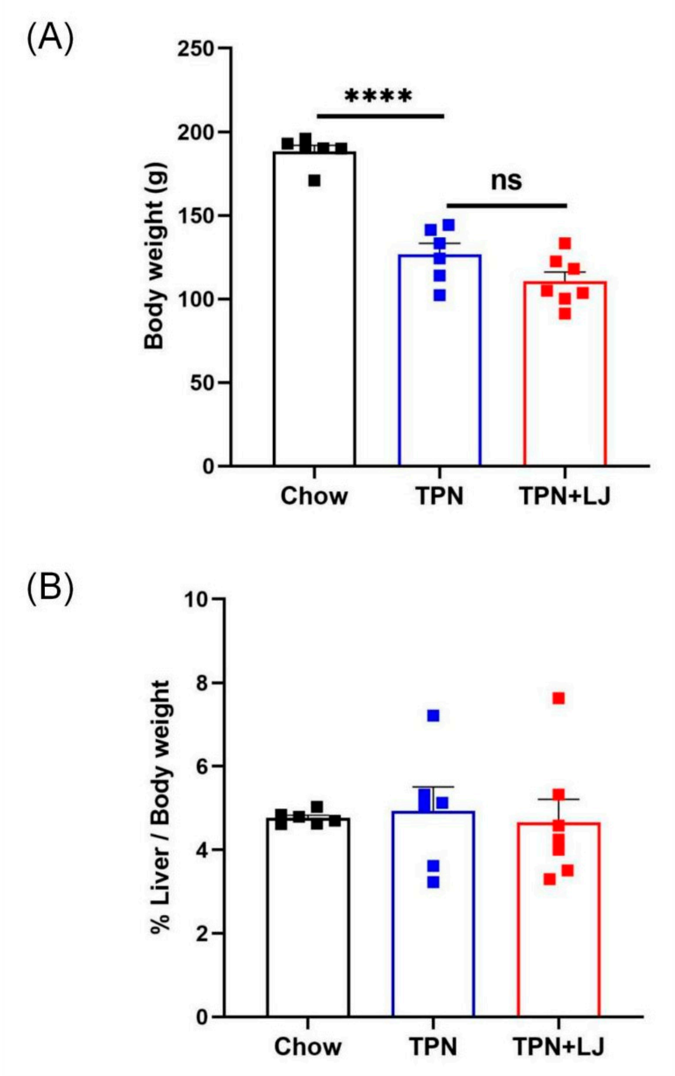

Supplementary Figure S1. The body weight (A) and ratio of liver weight to body weight (B) of rats from the control, TPN and TPN+*L. johnsonii* (LJ) groups. **** *P* < 0.0001, ns: not significant. n=6-7 per group.

Table S1. Summary of liver and serum bile acids composition in rats from the control, TPN and TPN+*L. johnsonii* (LJ) groups.

Bile acid	Percentage (%)			
	Control group (n = 6)	TPN group (n = 7-13)	TPN+LJ group (n = 4-7)	*P* value
**Liver (μg/g)**				
Primary bile acids				
αMCA	0.195	0.298	0.110	0.321
βMCA	2.079	1.782	0.883	0.155
βCA	0.120	0.528	0.341	0.114
CA	0.193	1.221	0.375	**0.009**
CDCA	0.055	0.239	0.084	0.202
GCA	9.229	31.372	6.739	**<0.001**
GCDCA	0.949	4.974	1.045	**0.002**
TαMCA	7.932	2.402	6.882	**<0.001**
TβMCA	44.922	8.555	30.184	**<0.001**
TCA	23.681	10.366	28.573	**<0.001**
TCDCA	6.291	3.854	15.535	**<0.001**
Secondary bile acids				
DCA	0.029	0.269	0.041	0.172
GDCA	0.212	7.694	0.560	**<0.001**
GHDCA	0.173	13.179	0.229	**<0.001**
GUDCA	0.493	1.165	0.557	0.060
HDCA	0.039	1.683	0.048	**0.017**
TDCA	1.057	4.397	4.914	0.051
THDCA+TUDCA	2.030	4.869	2.076	**0.004**
TLCA	0.106	0.394	0.425	**0.012**
Other bile acids	0.215	0.759	0.403	**0.025**
**Serum (μg/ml)**				
Primary bile acids				
αMCA	14.239	3.546	8.602	**0.003**
βMCA	16.429	17.166	15.121	0.832
CA	33.619	22.846	21.395	0.179
CDCA	6.417	2.807	9.297	**0.019**
GCA	1.609	6.456	3.474	0.115
GCDCA	0.284	0.929	0.954	0.361
GDCA	0.111	1.573	0.378	0.116
TαMCA	1.234	1.779	9.662	0.252
TβMCA	0.416	3.333	1.796	0.628
TCA	0.996	2.176	9.331	**0.026**
Secondary bile acids				
DCA	2.072	3.158	2.982	0.539
GHDCA	0.716	5.558	0.139	**0.007**
GUDCA	0.024	0.143	0.143	0.305
HDCA	17.360	22.663	1.745	**0.003**

LCA	0.085	0.019	1.339	**<0.001**
TDCA	0.187	0.317	0.865	**0.048**
UCA	0.533	1.375	4.066	0.261
Other bile acids	3.669	4.153	8.712	**0.035**

Notes: The percentage refers to the average percentage of individual bile acids in total bile acids; LJ, *Lactobacillus johnsonii*; TPN, total parenteral nutrition; αMCA, α-muricholic acid; βMCA, β-muricholic acid; βCA, β-cholic acid; CA, cholic acid; CDCA, chenodeoxycholic acid; GCA, glycocholic acid; GCDCA, glycochenodeoxycholic acid; TαMCA, tauroα-muriholic acid; TβMCA, tauroβ-muriholic acid; TCA, taurocholic acid; TCDCA, taurochenodeoxycholic acid; DCA, deoxycholic acid; GDCA, glycodeoxycholicacid; GHDCA, glycohyodeoxycholic acid; GUDCA, glycoursodeoxycholic acid; HDCA, hyodeoxycholic acid; TDCA, tauroursodeoxycholic acid; THDCA+TUDCA, taurohyodeoxycholic acid and tauroursodeoxycholic acid; TLCA, taurolithocholic acid; GDCA, glycodeoxycholicacid; LCA, lithocholic Acid; UCA, ursocholic acid.
